# Traumatic bone cyst of the mandible: a review of 26 cases

**DOI:** 10.1590/S1808-86942012000200004

**Published:** 2015-10-20

**Authors:** Paulo Ricardo Saquete Martins-Filho, Thiago de Santana Santos, Vanessa Lessa Cavalcanti de Araújo, Joanes Silva Santos, Emanuel Sávio de Souza Andrade, Luiz Carlos Ferreira da Silva

**Affiliations:** a Master's degree in health science, Nucleus for Graduate Studies in Medicine, Federal University of Sergipe (NPGME/UFS) (Doctoral student in health science, NPGME/UFS); b Master's degree in oral and maxillofacial surgery and trauma, Dentstry School of Pernambuco (FOP/UPE) (Doctoral student in oral and maxillofacial surgery, Dentstry School of Ribeirao Preto – FORP/USP); c Dental surgeon, trained at the Dentstry School of Pernambuco (FOP/UPE) (Dental surgeon); d Master's degree in oral and maxillofacial surgery and trauma, Federal University of Rio de Janeiro (UFRJ) (Coordinator of the oral surgery course, Center for Further Studies, Sergipe (CAP-SE)); e Doctoral degree in oral pathology, Federal University of Rio Grande do Norte (UFRN) (Professor of oral pathology, Dentstry School of Pernambuco – FOP/UPE); f Doctoral degree in oral and maxillofacial surgery and trauma, Dentstry School of Pernambuco (FOP/UPE) (Professor in the Dentstry Department and the NPGME/UFS). Faculdade de Odontologia de Pernambuco (FOP/UPE) – Dentstry School of Pernambuco. Universidade Federal de Sergipe (UFS) – Federal University of Sergipe

**Keywords:** bone cysts, mandible, nonodontogenic cysts

## Abstract

The traumatic bone cyst is characterized by the presence of an asymptomatic sinus devoid of epithelial lining, which is rarely found in the jaws.

**Objective:**

To describe the clinical, surgical and radiographic findings of traumatic bone cysts.

**Material and Method:**

A retrospective study was made of patients diagnosed with traumatic bone cysts at an oral pathology department from 1992 to 2007. Data on the clinical, radiographic and surgical complications were gathered.

**Results:**

Twenty-six cases of traumatic bone cyst were diagnosed in 15 years; 17 were male and 09 were female. Most patients were within first two decades of life and had no pain or history of trauma in the affected area. The multilocular pattern was observed in only seven cases, its radiographic appearance suggests a tumor. Air was found inside the lesion in about 70% of cases; serous fluid with blood and blood only were uncommon within the lesions.

**Conclusion:**

A higher prevalence in young patients, absence of a history of trauma, and a small number of lesions containing serous fluid with blood reflects the need to discuss the true pathogenesis of traumatic bone cysts.

## INTRODUCTION

Traumatic bone cysts may be characterized by the presence of an asymptomatic cavity in bone with no epithelial lining. Traumatic bone cysts were described in 1929;^1^ they are commonly found in the metaphysis of long bones, but are rare in the jaws^2^.

Traumatic bone cysts may be classified as unicameral^3^, simple^4^, solitary^5^, hemorrhagic^6^, or idiopathic^7^. They are usually asymptomatic and appear on routine radiographies. Because of a lack of unique clinical and radiographic features, it is important to establish the differential diagnosis between traumatic bone cysts and other bone lesions of the jaws – especially translucent lesions. The purpose of this study was to describe the clinical, surgical, and radiographic features of traumatic bone cysts that were diagnosed at an oral pathology center during a 15-year period.

## MATERIAL AND METHODS

A retrospective study was made of patients that had been diagnosed with traumatic bone cysts at an oral pathology center from 1992 to 2007. Two previously trained researchers reviewed the surgical reports in patient registries, panoramic radiographs, and hematoxylin-eosin stained slides to gather the following data:

(1) Clinical data: sex, age, pain, history of local trauma, anatomical site, presence of an enlarged mass;
(2) Radiographic data: number of lesions, whether locular, and diameter;
(3) Surgical: content of the pathologic cavity, and treatment.

Data were tabulated using the Bioestat 5.0 statistical package. Descriptive statistics were applied to obtain central tendency and dispersions measures. The institutional review board approved the study (protocol n°. 085/07.

## RESULTS

There were 26 cases of traumatic bone cysts were diagnosed in the 15-year period, comprising 0.58% of the total number of histopathology reports, and 13.5% of the bone lesions related to odontogenic tumors (Table 1). Table 2 summarizes the clinical, radiographic, and surgical findings of these traumatic bone cysts.

**Table 1:** Distribution of bone lesions related to odontogenic tumors diagnosed at an oral pathology clinic in a 15-year period.

Related Bone Lesions	n	%
Ossifying Fibroma	70	36.3
Fibrous Dysplasia of Bone	70	36.3
Traumatic Bone Cyst	26	13.5
Central Giant Cell Lesion	20	10.4
Aneurismatic Bone Cyst	05	2.5
Cherubism	02	1.0

Total	193	100

**Table 2:** Twenty-six cases of traumatic bone cysts: clinical, radiographic, and surgical features.

		Clinical Findings			Radiographic Findings			Findings at Surgery
Number	Sex/Age	Pain	History of trauma	Expansion	Site	Number of lesions/loculi	Diameter (cm)	Content
1	F/15	+	−	−	Mandibular body	One/Multilocular	6	Serous fluid
2	F/14	−	−	−	Mandibular body	One/Multilocular	3	Empty
3	F/14	−	−	+	Ramus	One/Multilocular	5	Serous-bloody fluid
4	M/16	−	−	−	Anterior area	One/Unilocular	3.5	Empty
5	M/16	−	−	−	Anterior area	One/Unilocular	3	Empty
6	M/16	−	−	−	Mandibular body	One/Unilocular	4	Empty
7	M/17	−	−	−	Mandibular body	One/Multilocular	1.5	Serous fluid
8	M/14	−	−	−	Anterior area	One/Unilocular	5	Empty
9	F/15	−	+	−	Mandibular body	One/Unilocular	4	Empty
10	M/17	−	−	−	Mandibular body	One/Unilocular	5	Empty
11	M/16	−	−	−	Mandibular body	One/Unilocular	2	Empty
12	M/22	−	−	−	Mandibular body	One/Unilocular	3.5	Empty
13	M/23	−	−	−	Anterior area	One/Unilocular	2	Serous fluid
14	F/70	+	−	−	Mandibular body	One/Multilocular	3	Empty
15	M/11	−	−	−	Anterior area	One/Unilocular	3	Empty
16	F/20	−	+	−	Anterior area	One/Unilocular	6	Empty
17	M/32	−	−	−	Anterior area	One/Unilocular	3	Empty
18	F/16	−	−	−	Anterior area	One/Unilocular	4	Serous-bloody fluid
19	M/20	−	−	−	Mandibular body	One/Unilocular	3	Empty
20	M/18	−	−	−	Mandibular body	One/Unilocular	3	Empty
21	M/10	−	+	+	Mandibular body	Multiple/Multilocular	6	Serous-bloody fluid
22	F/16	−	−	−	Mandibular body	One/Unilocular	8	Empty
23	M/17		−	−	Mandibular body	One/Unilocular	2	Empty
24	M/13	+	−	+	Mandibular body	One/Unilocular	4	Serous-bloody fluid
25	F/26	−	−	−	Mandibular body	One/Unilocular	4	Empty
26	M/23	−	+	−	Mandibular body	One/Multilocular	3.5	Serous-bloody fluid

### Clinical findings

Of 26 patients, 17 (65.4%) were male and nine were female (34.6%). The age ranged from 10 to 70 years; the mean was 19.5 years and the median was 16 years (Q1 = 15; Q3 = 20). Most cases were asymptomatic (88.5%); there was a history of trauma in only four patients (15.4%). All of the cases were on the mandible – 18 (69.2%) in the posterior area and eight (30.8%) in the anterior region. There was an enlarged mass in three cases (11.5%).

### Radiographic findings

**Figure 1:**
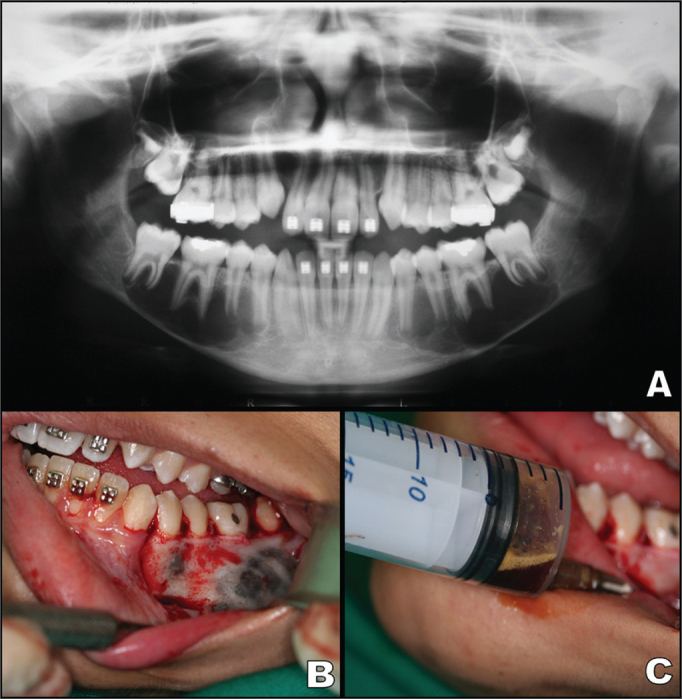
**A:** Panoramic radiograph showing a bilateral multilocular lesion with scalloping along the roots of teeth in the posterior area of the mandible. **B:** Intraoral view after making a mucoperiosteal flap to show bone expansion with hemorrhage. **C:** Aspiration biopsy showing serous-bloody fluid.

The lesions were solitary in most cases (96.1%); 19 (73.1%) were unilocular (Figure 1A). Seven cases were multilocular (26.9%), which gives the lesion the appearance of a tumor. The diameter on panoramic radiographs ranged from 1.5 to 8 cm; the median was 3.5 cm.

### Surgical findings

During surgery (Figure 1B), air was noted within the pathologic cavity in 18 cases (69.2%). There was serous-bloody fluid in five cases (19.2%) (Figure 1C) and serous fluid in three cases (11.6%). The treatment in all cases consisted of surgery to explore the cavity and curettage of the bone walls.

## DISCUSSION

Traumatic bone cysts are rare lesions of the jaws. They are classified by the World Health Organization^8^ as part of a group of bone lesions that include the ossifying fibroma, fibrous dysplasia of bone, *central giant cell lesions*, aneurismatic bone cysts, and cherubism. Although traumatic bone cysts were described at the beginning of the 20^th^ century, the pathogenesis remains unclear and speculative. The most accepted version at present is the traumatic-hemorrhagic theory, which suggests that lesions develop if intramedullary clots due to trauma do not undergo lysis or resolution^9^. This theory explains why traumatic bone cysts occur more often in young individuals (an age at which trauma occurs more often) and also explain the presence of blood within the cavity at the time of surgical exploration. Reports of trauma at the site of lesions and the presence of blood in the cavities, however, are uncommon, as seen in our sample. This opens the possibility that microtrauma of teeth and the alveolar ridge are involved in the pathogenesis of traumatic bone cysts^10^.

According to several authors, most cases of traumatic bone cysts present in young patients, although they may be detected at any age^11^, ^12^, ^13^; our findings were similar – there was a preference for the first two decades of life. MacDonald-Jankowski^12^ has stated that a reduced prevalence of traumatic bone cysts in older patients suggests that this lesion may be self-repairing – we believe this explanation is merely speculative. A few studies have reported no sex preference^13^, ^14^, but males predominated in our series. Zehetgruber et al.^15^ have reported a similar prevalence for extrafacial variants in young patients.

Traumatic bone cysts are considered almost exclusively mandibular lesions, with a preference for the posterior areas (body and ramus) – although the symphysis may also be a site^16^, ^17^. They rarely may be present in the maxilla^18^, although some studies have found a 25% incidence of cases in this bone^19^. Possibly its almost exclusive location in the mandible is related to its pathogenesis; the trauma-hemorrhage theory may be an explanation because the mandible, which has more cortical bone, repairs itself more slowly compared to the maxilla.

Most cases of maxillofacial traumatic bone cysts are asymptomatic and do not cause expansion of the cortical area – these cysts are diagnosed as accidental findings in routine radiographs^10^, ^12^, ^18^, ^20^. Except for three symptomatic cases in our series, all other were discovered in panoramic radiographs for conventional dental treatment, such as removal of third molars and orthodontic therapy. There was no bone expansion in about 92% of cases, which reflects the potential that this lesion has for developing in medullary spaces.

**Figure 2:**
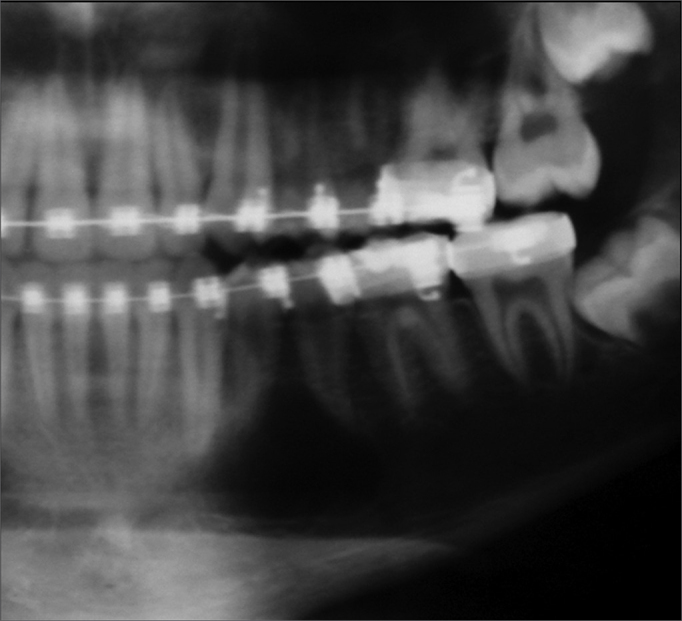
Panoramic radiograph showing a translucent unilocular image in the body of the left mandible. Radiotransparency reveals the classical scalloping around premolar apices.

**Figure 3:**
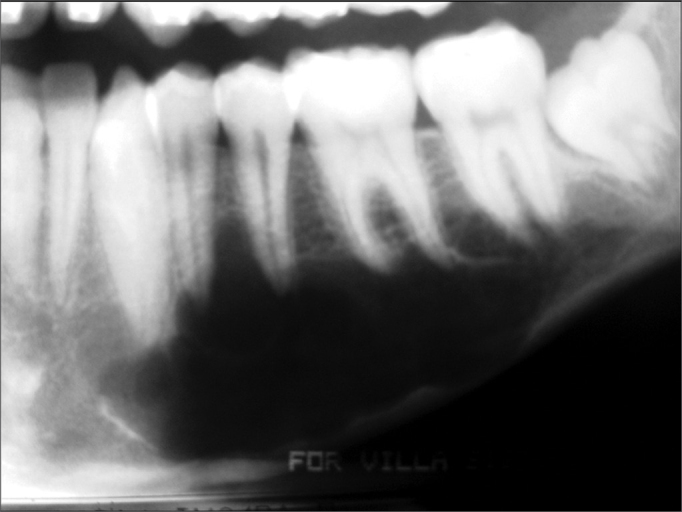
Multilocular traumatic bone cyst in the body of the left mandible; this image first suggested a tumor. The cortical plate is preserved.

Traumatic bone cysts generally show up as unilocular radiolucent areas in the posterior portion of the mandible; its margins are scalloped among dental roots^9^, ^10^, ^11^ (Figure 2). This radiographic pattern, however, may vary – the cyst may be multilocular, associated with unerupted/impacted teeth, and several cysts may be present in the same patient^17^ (Figure 3). Therefore, traumatic bone cysts should be part of the differential diagnosis of maxillary radiolucent lesions – together with dentigerous cysts, keratocystic odontogenic tumors, ameloblastomas, odontogenic myxomas, aneurismatic bone cysts, focal osteoporotic bone marrow defect, intraosseous vascular malformations, central giant cell lesions, among others. Although we found no association with unerupted/impacted teeth in our sample, the keratocystic odontogenic tumor and the ameloblastoma were the most frequently mentioned tumors in the initial diagnosis, especially when the radiographic image showed a multilocular cyst.

A few authors have reported the occurrence of multiple traumatic bone cysts and their association with fibrous/bony lesions – such as the florid cemento-osseous dysplasia – especially in older patients^20^, ^21^, ^22^, ^23^. Wakasa et al.^23^ have suggested that the florid cement-osseous dysplasia may precede traumatic bone cysts when these two conditions are associated, which suggests that disordered production of trabeculae in the former may obstruct lymphatic drainage and induce traumatic bone cyst formation. Our single bilateral case was not associated with other disorders – this patient was aged 10 years (Figures 1A, 1B, 1C).

The histology of traumatic bone cysts reveals only a connective tissue membrane lining the pathologic cavity, characteristic of pseudocysts. Cholesterol crystals, hemorrhagic foci, and osteoclasts may be found^10^, ^23^, ^24^. A final diagnosis of a traumatic bone cyst is almost invariably made at the time of surgery; the material available for histology is usually sparse because of the difficulty in removing the thin connective tissue membrane. Surgeons usually encounter an empty cavity, although there may be blood, serum, or both. Kuhmichel & Bouloux^20^ have noted that such content in the bone cavity may represent different stages in the development of traumatic bone cysts; we also believe this hypothesis is speculative.

The treatment of choice for traumatic bone cysts is surgery for curettage of the bone walls, which generally results in short-term healing^10^, ^18^, ^24^, ^25^. Recurrences are rare, and usually occur within three months of surgery. Cases of multiple cysts or those associated with florid cemento-osseous dysplasia have high recurrence rates – respectively about 71% and 75%^26^.

## CONCLUSION

In summary, although we found a predominance of traumatic bone cysts in males, the clinical and surgical findings in our sample concurred with other published studies. A higher prevalence of cases in the mandible and in young individuals, infrequent histories of trauma, and a paucity of lesions with serous-bloody content reflect a need to debate the true pathogenesis of traumatic bone cysts. In radiographs, the multilocular pattern comprises about one third of cases, and may simulate tumors in the jaws.
